# Two new African siblings of *Pulveroboletusravenelii* (Boletaceae)

**DOI:** 10.3897/mycokeys.43.30776

**Published:** 2018-12-12

**Authors:** Sylvestre A. Badou, André De esel, Olivier Raspé, Martin K. Ryberg, Atsu K. Guelly, Nourou S. Yorou

**Affiliations:** 1 Research Unit Tropical Mycology and Soil-Plant-Fungi Interaction, Laboratory of Ecology, Botany and Plant Biology, Faculty of Agronomy, University of Parakou, 03 BOX: 125, Parakou, Benin; 2 Meise Botanic Garden, Nieuwelaan 38, 1860 Meise, Belgium; 3 Fédération Wallonie-Bruxelles, Rue A. Lavallée 1, 1080 Bruxelles, Belgium; 4 Systematic Biology program, Department of Organismal Biology, Uppsala University, Norbyvägen 17D, 752 36 Uppsala, Sweden; 5 Département de Botanique et Écologie Végétale, Faculté des Sciences, Université de Lomé, BP1515 Lomé, Togo

**Keywords:** Boletales, Africa, *
Pulveroboletus
*, morphology, phylogeny, taxonomy

## Abstract

This paper sorts out the taxonomy of species affiliated with *Pulveroboletusravenelii* in the Guineo-soudanian and Zambezian woodlands of Africa. Morphological and genetic characters of African *Pulveroboletus* collections were studied and compared to those of North American and Asian species. A phylogenetic analysis showed that the African specimens form a subclade, sister to the Asian and American taxa. Although clamp connections have previously never been reported from *Pulveroboletus*, all specimens of the African subclade show very small clamp connections. Two new African species, *Pulveroboletusafricanus***sp. nov.** and *P.sokponianus***sp. nov.**, are described and illustrated. Comments concerning morphology and identification, as well as distribution and ecology, are given for both species.

## Introduction

Boletes belonging to *Pulveroboletus* Murrill are morphologically characterised by boletoid basidiomata with a pulverulent arachnoid veil. As originally indicated by Murrill (1909), this veil or cleistoblema *sensu*Clémençon (2012), is most often yellowish to vivid yellow and already present at a very early stage of development. Alterations broadening the circumscription of *Pulveroboletus* (Singer 1945, 1947, 1986), are not followed here as they have rendered the genus morphologically heterogeneous (Watling 2008) and polyphyletic (Binder and Hibbett 2006, Wu et al. 2014, Zeng et al. 2017). In its strict sense, *Pulveroboletus* holds few species, all of which are similar to the type, *Pulveroboletusravenelii* (Berk. & M.A. Curtis) Murrill. Based on molecular data, Raspé et al. (2016) stated that what is called *Pulveroboletusravenelii* outside North America belongs to a complex of different taxa. The name *P.ravenelii* has been used erroneously for lookalikes in Asia, Australia (Watling 2001, Horak 2011) and also Africa (Thoen and Ducousso 1989, Watling and Turnbull 1992, De Kesel et al. 2002, Yorou and De Kesel 2011, Vanié-Léabo et al. 2017, Kamou et al. 2017). So far, Raspé et al. (2016) and Zeng et al. (2017) have resolved part of the Asian complex around *P.ravenelii*, which now counts ten species. In a similar way, this paper aims to resolve and clarify the identity of some of the African, non-viscid, *Pulveroboletus* that have been kept under “Pulveroboletusaff.ravenelii”.

## Materials and methods

### Sampling, microscopy and morphology

Specimens were obtained from our own fieldwork or from herbarium specimens at our disposal. Protocols for field collecting, macroscopic description, drying and preservation follow Eyi Ndong et al. (2011). Codes and names of colours are according to the Methuen Handbook of Colour (Kornerup and Wanscher 1978). Microscopic structures were revived and examined in 5% potassium hydroxide (KOH) or in 10% ammonia with Congo Red. Measurements and drawings of microscopic structures were undertaken using an Olympus (BX51) compound microscope equipped with digital camera and drawing tube. Dimensions of microscopic structures are presented in the following format: (a–)b–c–d(–e), in which c represents the average, b = c − 1.96 * SD and d = c + 1.96 * SD and a and e are extreme values. *Q* is the length/width ratio based on at least 50 spores and is presented in the same format as spore dimensions (Eyi Ndong et al. 2011). Unless otherwise stated, herbarium specimens are deposited in BR. Duplicates from material from Togo are deposited in TOGO (Université de Lomé, Togo). Herbarium specimens from S. Badou (Benin) are deposited in UNIPAR (University of Parakou, Benin Republic). Abbreviations of herbaria follow Thiers (continuously updated). MycoBank (CBS-KNAW Fungal Biodiversity Centre, continuously updated) numbers are provided for the new species.

### DNA extraction, amplification and sequencing

Genomic DNA was isolated from CTAB-preserved tissues or dry specimens using a CTAB isolation procedure adapted from Doyle and Doyle (1990). The genes *atp*6, *tef*1 and *rpb*2 were amplified by PCR using the following primers: ATP6-1M40F and ATP6-2M (Raspé et al. 2016), EF1-983F and EF1-2218R (Rehner and Buckley 2005) and bRPB2-6F and bRPB2-7.1R (Matheny 2005). PCR products were purified by adding 1 U of Exonuclease I and 0.5 U FastAP Alkaline Phosphatase (Thermo Scientific, St. Leon-Rot, Germany) and incubated at 37 °C for 1 h, followed by inactivation at 80 °C for 15 min. Sequencing was performed by Macrogen Europe (The Netherlands) with PCR primers, except for *atp*6, for which universal primers M13F-pUC(-40) and M13F(-20) were used. For *tef*1, additional sequencing was performed with two internal primers, EF1-1577F and EF1-1567R (Rehner and Buckley 2005).

### Alignment and phylogeny inference

Sequences of *Pulveroboletus* species, including the type species *P.ravenelii*, along with sequences of various genera of the *Pulveroboletus* group (Wu et al. 2014) and three Leccinoideae species as outgroup were generated or retrieved from GenBank (Table 1). The sequences were assembled in GENEIOUS Pro v. 6.0.6 (Biomatters). All sequences were aligned using MAFFT (Katoh and Standley 2013) on the server accessed at http://mafft.cbrc.jp/alignment/server/ and introns in *rpb*2 and *tef*1 were identified based on the amino acid sequence of previously published DNA sequences. Maximum Likelihood (ML) phylogenetic tree inference was performed using RAxML (Stamatakis 2006) on the CIPRES web server (RAxML-HPC2 on XSEDE; Miller et al. 2009). The phylogenetic tree was inferred by a single analysis with four partitions (one for the exons of each gene and a fourth for the introns of *rpb*2 and *tef*1), using the GTRCAT model with 25 categories. Statistical support of nodes was obtained with 1,000 rapid bootstrap replicates.

**Table 1.** List of collections used for DNA analyses, with origin, GenBank accession numbers and references.

Species	Voucher	Origin	*atp6*	*tef1*	*rpb2*	Reference
* Baorangia pseudocalopus *	HKAS63607	China	–	KF112167	KF112677	Wu et al. 2014
* Baorangia pseudocalopus *	HKAS75739	China	–	KJ184570	KM605179	Wu et al. 2015
* Butyriboletus appendiculatus *	VDKO0193b	Belgium	MG212537	MG212582	MG212624	Vadthanarat et al. 2018
* Butyriboletus pseudoregius *	VDKO0925	Belgium	MG212538	MG212583	MG212625	Vadthanarat et al. 2018
* Butyriboletus pseudospeciosus *	HKAS63513	China	–	KT990743	KT990380	Wu et al. 2016
* Butyriboletus roseoflavus *	HKAS54099	China	–	KF739779	KF739703	Wu et al. 2014
* Butyriboletus roseopurpureus *	BOTH4497	USA	MG897418	MG897428	MG897438	Phookamsak et al. 2018
* Butyriboletus subsplendidus *	HKAS50444	China	–	KT990742	KT990379	Wu et al. 2016
Butyriboletus cf. roseoflavus	OR230	China	KT823974	KT824040	KT824007	Raspé et al. 2016
* Caloboletus calopus *	ADK4087	Belgium	MG212539	KJ184566	KP055030	Vadthanaratet al. 2018, Zhao et al. 2014a, 2014b
* Caloboletus inedulis *	BOTH3963	USA	MG897414	MG897424	MG897434	Phookamsak et al. 2018
* Caloboletus radicans *	VDKO1187	Belgium	MG212540	MG212584	MG212626	Vadthanarat et al. 2018
* Caloboletus yunnanensis *	HKAS69214	China	–	KJ184568	KT990396	Zhao et al. 2014a, Wu et al. 2016
* Cyanoboletus brunneoruber *	OR0233	China	MG212542	MG212586	MG212628	Vadthanarat et al. 2018
* Cyanoboletus pulverulentus *	RW109	Belgium	KT823980	KT824046	KT824013	Raspé et al. 2016
*Cyanoboletus* sp.	OR0257	China	MG212543	MG212587	MG212629	Vadthanarat et al. 2018
* Lanmaoa angustispora *	HKAS74752	China	–	KM605154	KM605177	Wu et al. 2015
* Lanmaoa asiatica *	HKAS63603	China	–	KM605153	KM605176	Wu et al. 2015
* Lanmaoa carminipes *	BOTH4591	USA	MG897419	MG897429	MG897439	Phookamsak et al. 2018
* Neoboletus brunneissimus *	HKAS50538	China	–	KM605150	KM605173	Wu et al. 2015
* Neoboletus brunneissimus *	OR0249	China	MG212551	MG212595	MG212637	Vadthanarat et al. 2018
* Neoboletus junquilleus *	AF2922	France	MG212552	MG212596	MG212638	Vadthanarat et al. 2018
* Neoboletus magnificus *	HKAS54096	China	–	KF112149	KF112654	Wu et al. 2014
* Neoboletus venenatus *	HKAS63535	China	–	KT990807	KT990448	Wu et al. 2016
*Pulveroboletusafricanus* (type)	ADK4650	Togo	KT823959	KT824025	KT823992	Raspé et al. 2016
* Pulveroboletus brunneopunctatus *	OR0147	China	MG897420	MG897430	MG897440	Phookamsak et al. 2018
* Pulveroboletus brunneopunctatus *	HKAS55369	China	–	KT990814	KT990455	Wu et al. 2016
* Pulveroboletus brunneopunctatus *	HKAS74926	China	–	KT990815	KT990456	Wu et al. 2016
* Pulveroboletus fragrans *	OR0673	Thailand	KT823977	KT824043	KT824010	Raspé et al. 2016
* Pulveroboletus macrosporus *	HKAS57628	China	–	KT990812	KT990453	Wu et al. 2016
*Pulveroboletussokponianu*s (type)	ADK4360	Togo	KT823957	KT824023	KT823990	Raspé et al. 2016
* Pulveroboletus sokponianus *	SAB0629	Benin	MH983001	MH983002	MH983003	This study
* Pulveroboletus ravenelii *	REH2565	U.S.A.	KU665635	KU665636	KU665637	Raspé et al. 2016
*Pulveroboletus* sp.	OR0282	China	MK058515	MK058518	MK058521	This study
*Pulveroboletus* sp.	OR0644	Thailand	MK058516	MK058519	MK058522	This study
*Pulveroboletus* sp.	OR0686	Thailand	MK058517	MK058520	MK058523	This study
* Retiboletus fuscus *	OR0231	China	MG212556	MG212600	MG212642	Vadthanarat et al. 2018
* Retiboletus griseus *	MB03-079	U.S.A.	KT823964	KT824030	KT823997	Raspé et al. 2016
* Rhodactina rostratispora *	SDBR-CMU-SV208	Thailand	MG212561	MG212606	MG212646	Vadthanarat et al. 2018
* Rubroboletus legaliae *	VDKO0936	Belgium	KT823985	KT824051	KT824018	Raspé et al. 2016
* Rubroboletus rhodosanguineus *	BOTH4263	USA	MG897416	MG897426	MG897436	Phookamsak et al. 2018
* Rubroboletus satanas *	VDKO0968	Belgium	KT823986	KT824052	KT824019	Raspé et al. 2016
* Rubroboletus sinicus *	HKAS56304	China	–	KJ619483	KP055031	Zhao et al. 2014a; Zhao et al. 2014b
* Rugiboletus brunneiporus *	HKAS83209	China	–	KM605144	KM605168	Wu et al. 2015
* Rugiboletus extremiorientalis *	HKAS76663	China	–	KM605147	KM605170	Wu et al. 2015
* Rugiboletus extremiorientalis *	OR0406	Thailand	MG212562	MG212607	MG212647	Vadthanarat et al. 2018
* Suillellus luridus *	VDKO0241b	Belgium	KT823981	KT824047	KT824014	Raspé et al. 2016
* Suillellus subamygdalinus *	HKAS53641	China	–	KT990841	KT990478	Wu et al. 2016
* Sutorius australiensis *	REH9441	Australia	MG212567	JQ327032*	MG212652	Halling et al. 2012*, Vadthanaratet al. 2018
* Sutorius eximius *	REH9400	U.S.A.	MG212568	JQ327029*	MG212653	Halling et al. 2012*, Vadthanaratet al. 2018

## Results

### DNA analyses

**Figure 1.**
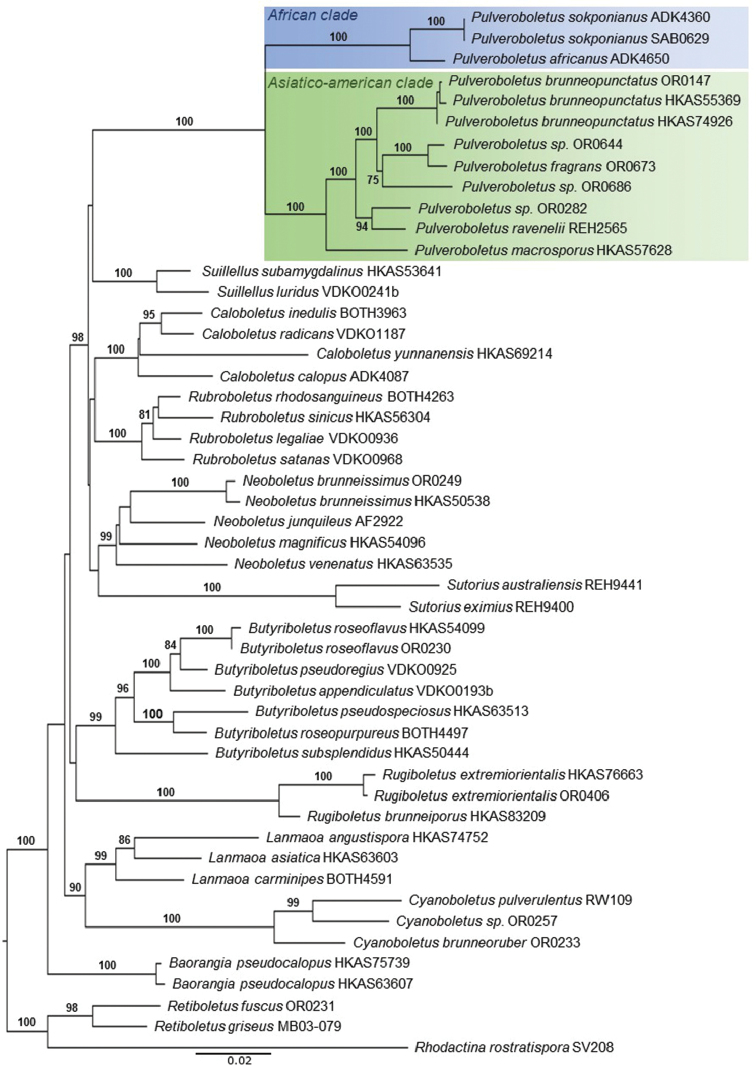
Maximum likelihood phylogenetic tree inferred from the three-gene dataset (*atp*6, *rpb*2, *tef*1), including *Pulveroboletusafricanus* sp. nov. and *Pulveroboletussokponianus* sp. nov and selected Boletaceae. The three Leccinoideae species (*Retiboletusfuscus* (Hongo) N.K. Zeng & Zhu L. Yang, *R.griseus* (Frost) Manfr. Binder & Bresinsky and *Rhodactinarostratispora* S. Vadthanarat, O. Raspé & S. Lumyong) were used as outgroup taxa. Bootstrap frequencies > 70% are shown above supported branches.

The alignment contained sequences from 50 specimens and was 2,649 characters long (TreeBase number 23416). In the phylogram obtained (Fig. 1), *Pulveroboletus* formed a highly supported clade (BS = 100%). Interestingly, the African species formed a highly supported sub-clade sister to the Asian and American species, which together formed another highly supported sub-clade.

### Taxonomy

#### Pulveroboletus africanus

Taxon classificationPlantaeBoletalesBoletaceae

De Kesel & Raspé sp. nov.

827970

Fig. 2a–f

##### Illustr.

Sharp (2011, page 41, ut Pulveroboletusaff.ravenelii).

##### Holotype.

Togo, Central Province, Fazao National Park, 08°44'31.9"N, 0°48'17.4"E, 6 June 2008, elev. 500 m, on the ground in a gallery forest with *Berliniagrandiflora* (Vahl) Hutch. & Dalziel, *Uapacaguineensis* Müll.-Arg. and *Pandanus* sp., leg. A. De Kesel, De Kesel 4650 (BR!, BR 5020165390056, duplicate in TOGO).

##### Etymology.

Epithet refers to its very wide distribution throughout tropical Africa.

##### Description.

Basidiomata medium-sized, covered by a general veil when young. Pileus 60–70 mm diam., at first broadly convex then pulvinate to plano-convex, upper layer dark brown (6E6–6F6), dry, mat, tomentose to felty, very soon cracking, becoming tomentose-scaly, bright yellow (2A4–5) in the deeper layers, predominantly yellow with age; scales appressed, slightly fibrillose, leather brown to greyish-brown (6E4–5), thicker and darker in the centre, thinner, paler and more diffused towards the margin; margin at first incurved, appendiculate with age, vivid yellow, beset with sulphur-yellow pulverulent material. Hymenophore tubulate, separable, straight to slightly sinuate, almost free around the stipe or depressed and then only slightly decurrent with a tooth; tubes up to 10 mm long, yellow to greyish-yellow (1B3), cyanescent when cut; pores regular, mostly round or slightly angular, slightly elongated around the stipe, small (14–16/mm), yellow (1A2–2A2), with age greyish-brown (5E4–6), cyanescent. Stipe cylindrical, 60–80 × 8–12 mm, central, solid, uppermost part vivid yellow (2A4–5), often with some reddish fibrils and smooth, lower part sheathed from the base up with a mat, dry, fibrillose-cottony, greyish to brownish-grey (5–6EF3–4) layer, the latter cracking transversally, forming brownish-grey to olive brown patches (4DE4–6), first exposing a greyish-white layer, then the bright yellow deeper layers; ring at first prominent, loose membranous-cottony, vivid yellow (2AB3–5), covering the pores, later tearing, leaving fibrillose to membranous material on both pileus margin and upper third of the stipe, pulverulent, becoming greenish from spores.

Context yellowish-white in the pileus, marmorated yellow (1–2A2) – yellowish-white in the stipe, yellow towards the base of the stipe, cyanescent in all parts. Basal mycelium and rhizomorphs relatively thick, yellowish-white (2A2) to yellow. Odour strong fungoid. Taste not recorded. Spore print greenish-olive (fresh 3D4 in Rammeloo 5720).

Macrochemical reactions: tubes brown to reddish-brown with KOH and NH_4_OH (in collections Rammeloo 5922 and De Kesel 2163).

Spores (8.4)8.6–9.5–10.3(–10.6) × (4.5)4.5–4.9–5.3(5.4) µm, Q = (1.77)1.79–1.93–2.07(2.09), broadly ellipsoid, smooth, pale yellowish-brown in 5% KOH and Melzer’s reagent, thin-walled, inamyloid. Basidia 4-spored, 22–35 × 8–12 µm, clavate, hyaline, sterigmata up to 3–4 µm long, without clamp connection. Cheilocystidia abundant, cylindrical to narrowly fusiform, (31.9–)32.1–42.6–53(–48.8) × (6.1–)5.6–7.2–8.7(–8.6) µm, thin-walled, hyaline. Pleurocystidia similar to cheilocystidia, not abundant. Hymenophoral trama subregular, with poorly defined mediostratum. Pileipellis a tomentum composed of irregularly arranged hyphae, the latter cylindrical, of similar shape, 3.8–5.1(6.5) µm wide, slightly thick-walled (0.5 µm), with brownish intracellular pigment (persistent in 5% KOH), smooth, with pulverulence in places. Stipitipellis a tomentocutis composed of elements similar to the pileipellis. Partial veil composed of cylindrical hyphae, 3–6 µm wide, thin-walled and smooth. Clamp connections present in pileipellis tissue, small, frequent.

##### Additional collections.

BENIN, Atacora Province, Kota, 10°12.680'N, 1°26.723'E, 30 Aug. 1997, 490 m a.s.l., gallery forest with *Berliniagrandiflora* and *Uapacaguineensis*, De Kesel 2023 (BR 5020074869827); ibidem, 10°12.699'N, 1°26.786'E, 17 Jun. 2000, 490 m a.s.l., De Kesel 2824 (BR 5020126377836); ibidem, 10°12.665'N, 1°26.750'E, 30 Jun. 2002, 510 m a.s.l., De Kesel 3500 (BR 5020152209163); Borgou Province, Wari Maro, 9°09.884'N, 2°09.595'E, 20 Jun. 1998, 300 m a.s.l., savannah woodland with *Isoberliniadoka* Craib & Stapf and *Uapacatogoensis* Pax, De Kesel 2163 (BR 5020112674574); BURUNDI, Bururi Province, Mugara, 04°02'S, 29°31'E, 16 Nov. 1978, 1050 m a.s.l., *Brachystegia* woodland, Rammeloo 5720 (BR 5020019368651);) ; ibid., 18 Nov. 1978, Rammeloo 5788 (BR 5020019374713); ibid., 20 Nov. 1978, Rammeloo 5811 (BR 5020032463654); ibid., 29 Nov. 1978, 950–1050 m a.s.l., *Brachystegia* woodland, Rammeloo 5922 (BR 5020019378759); ibid., Rumonge-Mutambara, 4°0.756'S, 29°29.599'E, 11 Jan. 2011, 950 m a.s.l., miombo woodland with *Brachystegiautilis* Burtt Davy & Hutch. and *B.bussei* Harms, Degreef 673 (BR); GUINEA, Labé Prefecture, Fouta Djalon, N of Tountourou, 13 Jul. 1988, 1000 m a.s.l., mountain woodland with *Uapacachevalieri* Beille, Thoen 7977 (BR 5020003130264); DR CONGO, Upper Katanga Province, near Kisangwe, Mikembo sanctuary, 11°28.790'S, 27°40.367'E, 2 Feb. 2012, 1170 m a.s.l., miombo woodland with *Julbernardiaglobiflora* (Benth.) Troupin and *Brachystegia* spp, De Kesel 5026 (BR 5020212174363V); MALAWI, Nkhata bay district, Chisosira, 16 miles south of Chinteche, 3 Jan. 1978, woodland with *Brachystegiaspiciformis* Benth., 450 m a.s.l., E. Tybaert 141 (BR 5020019389861, dupl. GENT); MOZAMBIQUE, Nambula Province, Natala, Reserva de Mecuburi, 27 Jan. 2011, leg. M. Härkönen, Marja Härkönen 201131 (H 7016064); TOGO, Central Province, Kparatao (towards Bassar), 09°11.630'N, 0°59.134'E, 14 Jul. 2007, 580 m a.s.l., miombo woodland with *Uapacatogoensis* and *Monoteskerstingii* Gilg., De Kesel 4359 (BR 5020163710719, duplicate in TOGO); ZIMBABWE, Midlands Province, Mtao Forest, 19°22.081'S, 30°40.383'E, 11 Feb. 1999, 1500 m a.s.l., extensively grazed miombo woodland, under *Brachystegiaspiciformis*, De Kesel 2453 (BR 5020112623060).

##### Ethnomycological data.

except for Mozambique and Zimbabwe, no local names or uses were collected. The local name in Mozambique (in Makua) is *Ettuli ya Khapa* (coll. Marja Härkönen 201131), which means tortoise shell. The local name in Zimbabwe (in chiShona) is *dindindi java* (Sharp 2011). The species is not used for consumption.

**Figure 2.**
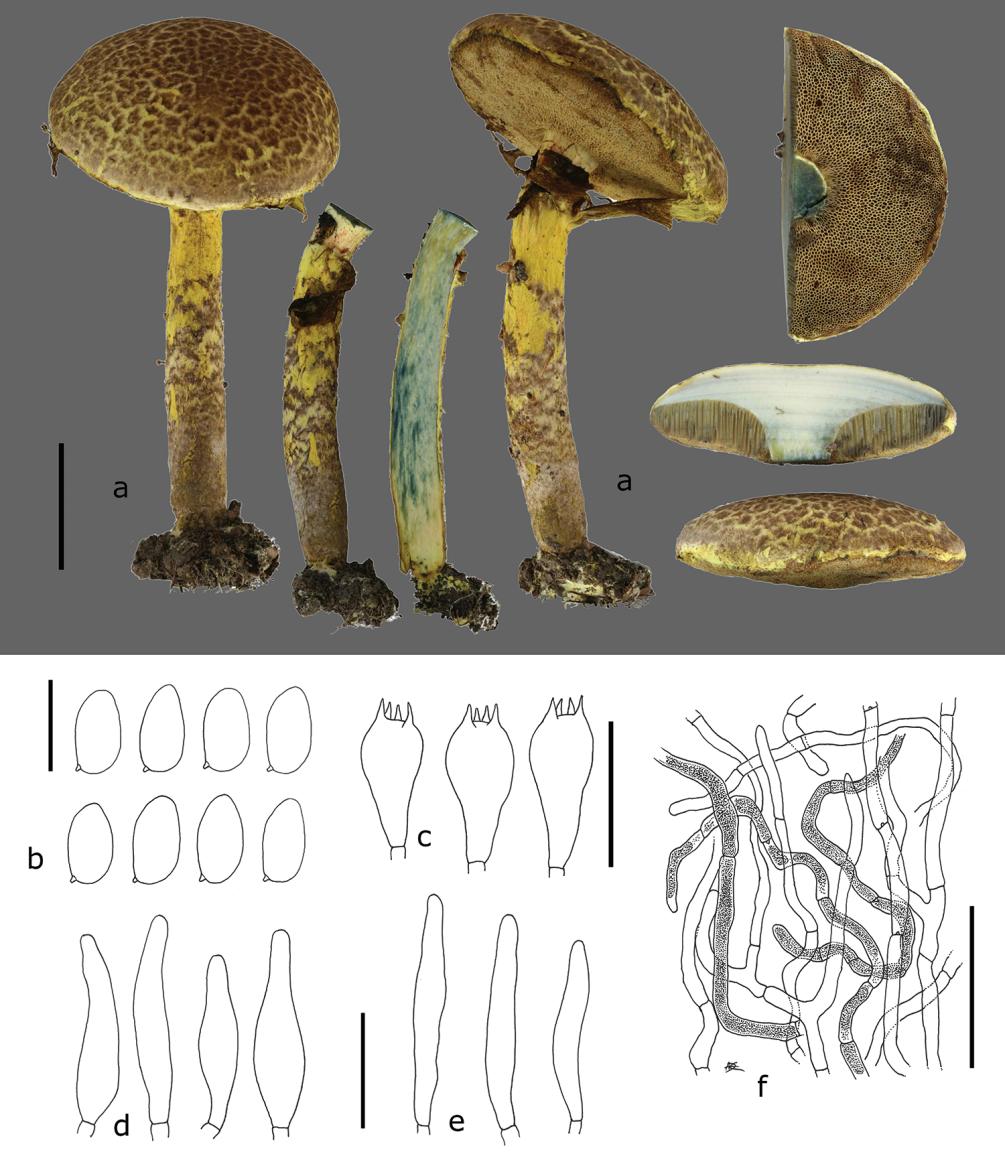
*Pulveroboletusafricanus* (De Kesel 4650, holotype): **a** basidiomes **b** basidiospores **c** basidia **d** cheilocystidia **e** pleurocystidia **f** pileipellis hyphae with intracellular pigments and tiny clamps. Scale bars: 25mm (**a**), 10µm (**b**), 25µm (**c**), 25µm (**d, e**), 50µm (**f**).

#### Pulveroboletus sokponianus

Taxon classificationPlantaeBoletalesBoletaceae

Badou, De Kesel, Raspé & Yorou sp. nov.

827974

Fig. 3a–g

##### Illustr.

Yorou and De Kesel (2011, fig 5.10, ut *Pulveroboletusravenelii*).

##### Holotype.

Togo, Central Province, Kparatao (towards Bassar), 09°11.630'N, 0°59.134'E, 14 July 2007, alt. 580 m, woodland on a slope with *Isoberliniadoka*, *Uapacatogoensis* and *Monoteskerstingii*, leg. A. De Kesel, De Kesel 4360 (BR!, BR 5020163695566, duplicate in TOGO).

##### Etymology.

in honour of the late Prof. Dr. Ir. Nestor Sokpon, esteemed colleague of the University of Parakou (Benin), for his various contributions to the understanding of woodland ecology and regeneration.

##### Description.

Basidiomata medium-sized, wrapped in a greenish-yellow (1A2–3) general veil when young. Pileus 40–55(60) mm diam., at first hemispherical to convex, then pulvinate or plano convex, upper layer pale yellow (1A2–4) to greenish-grey (1BC3–4), not cyanescent, dry, mat, tomentose to felty, becoming subtly to inconspicuously cracked, greenish-yellow (1A2–3) in the deeper layers; scales subtle, flat, slightly felty, greenish-grey (1BC3–4), darker in the centre, diffused towards the margin; margin at first incurved, appendiculate with age, greenish-yellow. Hymenophore tubulate, separable, straight to slightly sinuate, depressed around the stipe; tubes up to 7 mm long, yellow to greyish-yellow (1B3), cyanescent when cut; pores regular, mostly round or slightly angular, slightly elongated around the stipe, small (13–16/mm), pale yellow (1A2–2A2), cyanescent. Stipe cylindrical, 42–60 × 6–7(9) mm, central, solid, uppermost part yellowish-white (1A2–3), smooth, lower part sheathed with a mat, dry, fibrillose-cottony, thick, yellowish-white to pale yellow (1A2–4) or pale greenish-grey (1BC3–4) layer, the latter rather tearing than cracking in subtle fibrils; ring at first woolly, cottony, pale greenish-yellow (1A2–4), then collapsing, leaving diffuse remains on pileus margin and stipe, sometimes pulverulent. Context whitish to whitish-yellow in the pileus, gradually yellowish-white (1A2) towards the base of the stipe. Slightly and slowly cyanescent, except in the base of the stipe. Basal mycelium and rhizomorphs usually white. Odour fungoid, when fresh like *Lepistanuda* (in collection De Kesel 1979). Spore print and macrochemical reactions not obtained.

Spores (8.5–)8.5–9.3–10.2(–10.5) × (4.4–)4.3–4.9–5.4(–5.6) µm, Q = (1.76)1.74–1.92–2.1(–2.14), broadly ellipsoid, smooth, pale yellowish-brown in 5% KOH and Melzer’s reagent, thin-walled, inamyloid. Basidia 4-spored, 21–32 × 8–12 µm, clavate, hyaline, sterigmata 3–4 µm long, without clamp connection. Cheilocystidia abundant, fusiform to clavate, (36.8–)34.1–42.7–51.4(–52.8) × (6.6–)7.7–9.7–11.7(–11) µm, thin-walled, with yellow intracellular pigment (persistent in NH_4_OH). Pleurocystidia similar to cheilocystidia, not abundant. Hymenophoral trama divergent, with regular mediostratum. Pileipellis a tomentum, composed of irregularly intertwined hyphae of similar shape, cylindrical, 3.3–5.1(6.2) µm wide, entirely hyaline, smooth, with small clamps. Stipitipellis a tomentum composed of elements similar to the pileipellis. Partial veil with cylindrical hyphae, 3–6 µm wide, thin-walled, smooth. Clamp connections small, frequent in the pileipellis.

##### Additional collections.

BENIN, Atacora Province, Natitingou, Kota falls, 10°12.680'N, 1°26.723'E, 23 Aug. 1997, 520 m a.s.l., savannah woodland with *Isoberlinia*, A. De Kesel 1979 (BR 5020074831442); ibidem, 10°12.555'N, 001°26.793'E, 26 Jun. 2004, 480 m a.s.l., forest gallery with *Berliniagrandiflora* and *Uapaca* sp., A. De Kesel 3769 (BR 5020152060610); ibidem, Kouandé, 10°17.159'N, 1°40.890'E, 25/09/2000, 470 m a.s.l., savannah woodland with *Isoberliniatomentosa* (Harms) Craib & Stapf, A. De Kesel 2942 (BR 5020129153468); ibidem, Borgou Province, Doguè, 9°07.249'N, 1°54.839'E, 10/10/2001, 350 m a.s.l., savannah woodland with *Afzeliaafricana* S.M. and *Isoberliniadoka*, A. De Kesel 3213 (BR 5020149693227); ibidem, Borgou Province, Okpara, 9°14.669'N, 2°43.377'E, 9 Aug. 2017, 360 m a.s.l., savannah woodland with *Isoberliniadoka*, S. Badou 0629 (UNIPAR); ibidem, Tamarou (forêt classée de N’dali), 9°44.680'N, 2°41.544'E, 31 Jul. 2017, 390 m a.s.l., savannah woodland with *Isoberliniadoka*, S. Badou 0624 (UNIPAR); ibidem, 4 Aug. 2017, 390m a.s.l., S. Badou 0625 (UNIPAR); ibidem, Wako, 9°09.457'N, 2°05.599'E, 11/09/2001, 300 m a.s.l., savannah woodland with *Isoberliniadoka*, *Uapacatogoensis* and *Burkeaafricana* Hook., A. De Kesel 3132 (BR 5020149809413); ibidem, Wari Maro, 9°10.038'N, 2°09.931'E, 20 Aug. 1997, 310 m a.s.l., savannah woodland with *Isoberliniadoka*, A. De Kesel 1943 (BR 5020074934501); ibidem, 9°09.884'N, 2°09.595'E, 22 Jun. 1998, 310 m a.s.l., savannah woodland with *Isoberliniadoka*, A. De Kesel 2183 (BR 5020112693766); ibidem, 9°08.110'N, 2°10.215'E, 09/10/2001, 290 m a.s.l., savannah woodland with *Isoberliniadoka* and *Uapacatogoensis*, A. De Kesel 3188 (BR 5020149726550); ibidem, 9°09.900'N, 2°09.511'E, 23/09/2001, 310 m a.s.l., savannah woodland with *Isoberliniadoka* and *Uapacatogoensis*, A. De Kesel 3237 (BR 5020149751804); ibidem, 9°10.027'N, 2°10.848'E, 16 Jun. 2002, 340 m a.s.l., savannah woodland with *Isoberliniadoka* and *Uapacatogoensis*, A. De Kesel 3411 (BR 5020152133376); ibidem, Collines Province, Toui-Kilibo, 8°32.746'N, 2°40.424'E, 19 Jul. 2017, 320 m a.s.l., savannah woodland with *Isoberliniadoka* and *I.tomentosa*, S. Badou 0519 (UNIPAR); ibidem, 5 Jul. 2017, S. Badou 0617 (UNIPAR); ibidem, 13 Jul. 2017, S. Badou 0621 (UNIPAR); ibidem, Donga Province, Bassila, 8°57.319'N, 1°38.391'E, 14 Jun. 2002, 380 m a.s.l., savannah woodland with *Berliniagrandiflora*, A. De Kesel 3403 (BR 5020152245529); ibidem, 8°59.516'N, 1°38.261'E, 26 Jun. 2002, 370 m a.s.l., gallery forest with *Berliniagrandiflora*, *Elaeisguineensis* Jacq. and *Raphia* sp., A. De Kesel 3467 (BR 5020152045464); ibidem, 9°0.073'N, 001°39.318'E, 17 Jun. 2004, 380 m a.s.l., gallery forest with *Berliniagrandiflora*, A. De Kesel 3668 (BR 5020157041959); ibidem, Penessoulou (south), 9°9.688'N, 1°34.793'E, 4 Jul. 2017, 380 m a.s.l., small gallery forest with *Isoberliniadoka*, S. Badou 0613 (UNIPAR); ibidem, 11 Aug. 2017, S. Badou 0630 (UNIPAR); ibidem, 22 Aug. 2017, S. Badou 0631 (UNIPAR); ibidem, Zou Province, Ouèssè, Gbadji forest (West side of the slope), 7°57.152'N, 001°58.095'E, 13 Jun. 2004, 310 m a.s.l., savannah woodland with *Isoberliniadoka*, *Burkeaafricana*, A. De Kesel 3593 (BR 5020157206662). TOGO, Central Province, Fazao (Parc National), 08°42.150'N, 0°46.383'E, 16 Jun. 2011, 520 m a.s.l., savannah woodland with *Afzeliaafricana*, A. De Kesel 4910 (BR 5020212173335V); ibidem, 08°43.963'N, 0°47.674'E, 16 Jul. 2007, 510 m a.s.l., savannah woodland with *Isoberliniadoka* and *Uapacatogoensis*, A. De Kesel 4382 (BR 5020163713741); ibidem, 08°38.737'N, 0°46.010'E, 17 Jul. 2007, 550 m a.s.l., gallery forest with *Berliniagrandiflora*, A. De Kesel 4393 (BR 5020163839069); ibidem, 08°43.145'N, 0°46.332'E, 20 Jul. 2007, 560 m a.s.l., savannah woodland on gravelly soil, with *Uapacatogoensis*, A. De Kesel 4469 (BR 5020163803671); ibidem, 08°40.872'N, 0°45.487'E, 04 Jun. 2008, 680 m a.s.l., savannah woodland with *Isoberliniadoka* and *Uapacatogoensis*, A. De Kesel 4625 (BR 5020165412277).

##### Ethnomycological data.

Except for Benin, no local names or uses were collected. The local name in Nagot language is *osousou eti* (coll. De Kesel 2183) and this species is not eaten.

**Figure 3.**
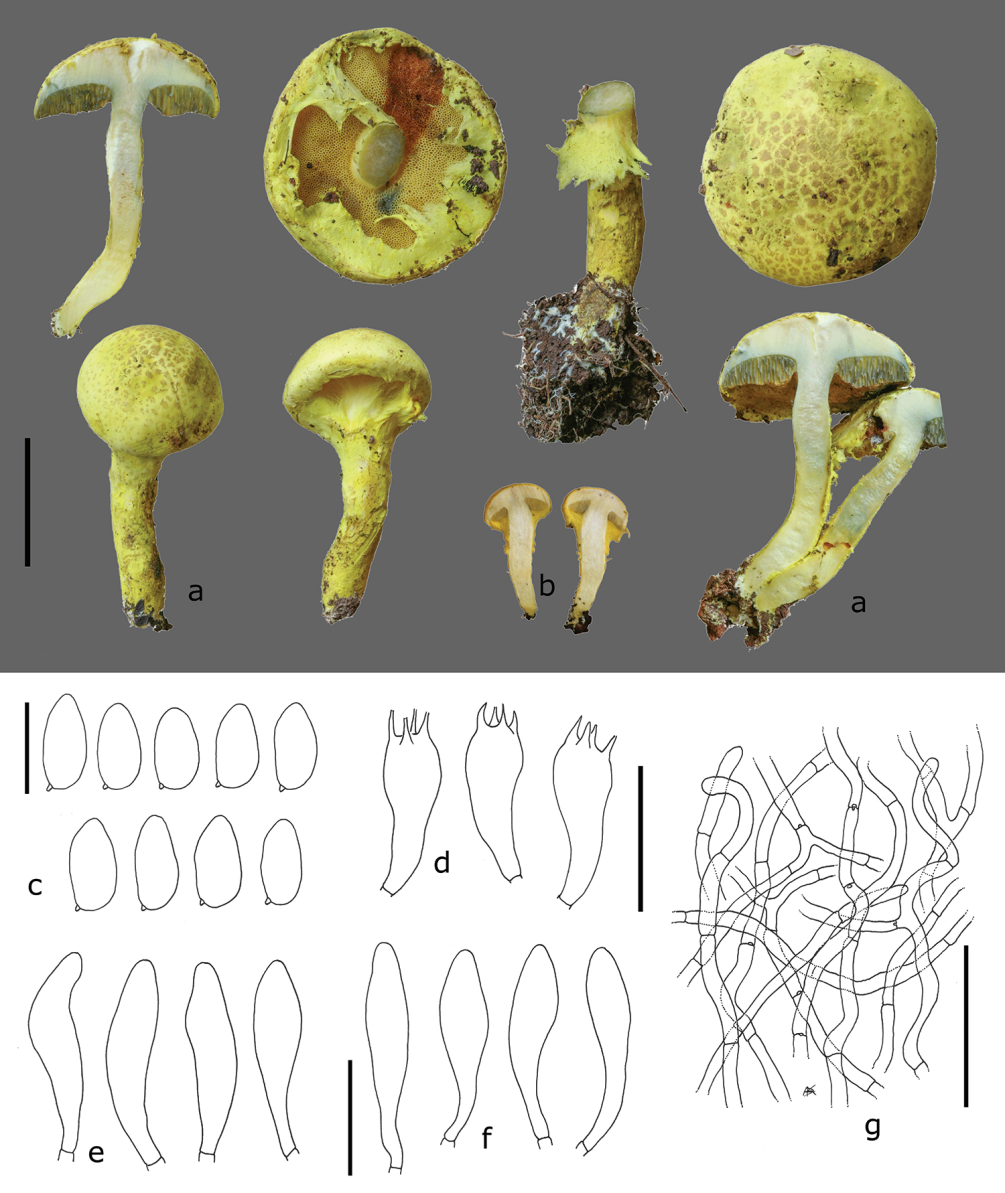
*Pulveroboletussokponianus* (**a, c–g** De Kesel 4360, holotype, **b** De Kesel 3593): **a** basidiomes **b** very young basidiomes **c** basidiospores **d** basidia **e** cheilocystidia **f** pleurocystidia **g** pileipellis with tiny clamps. Scale bars: 25mm (**a, b**), 10µm (**c**), 25µm (**d**), 25µm (**e, f**), 50µm (**g**).

## Discussion

### Species delimitation

The African collections represent two separate species, *Pulveroboletusafricanus* sp. nov. and *P.sokponianus* sp. nov., both macroscopically similar to *Pulveroboletusravenelii*. In the latter, however, the disc becomes reddish-orange to reddish-brown at maturity and it grows in temperate conifer woods (Bessette et al. 2016), montane Quercus forests in Costa Rica (Halling and Mueller 2005) and Colombia (Franco-Molano et al. 2000) and Pine-oak forests in the Dominican Republic/Belize (Ortiz-Santana et al. 2007). The phylogenetic analysis showed that the African specimens form a well-supported subclade within *Pulveroboletus*, sister to the Asian and American taxa. Although clamp connections have previously been reported to be absent in *Pulveroboletus* (Watling 2008, Zeng et al. 2017), all specimens of the African subclade show very small clamp connections.

Macroscopically, both African taxa can be distinguished based on the colour of the scales on the pileus and the stipe, being brown in *P.africanus* and greenish-grey or yellow in *P.sokponianus*. In *P.africanus*, the basal mycelium and context in the base of the stipe is generally yellow whereas it is whitish to whitish-yellow in *P.sokponianus*. While bluing of the context depends on the freshness and the maturity of the basidiomes, it seems more pronounced in *P.africanus*. Although cystidia have been reported to be rather constant and of little use to separate Asian taxa (Zeng et al. 2017), this seems to be true for the spores of the African taxa, but not for cystidia. In *P.africanus*, the cheilocystidia are hyaline and narrowly fusiform, whereas they are broadly fusiform and yellow pigmented in *P.sokponianus*. Further striking characters of distinction is the brownish intracellular pigmentation in the hyphae of the pileal and stipital scales, present in *P.africanus* but absent in *P.sokponianus*.

Young basidiomes of *Pulveroboletussokponianus* are strongly reminiscent of the Asian *P.brunneopunctatus* G. Wu & Zhu L. Yang, but the latter has a viscid veil and smaller cheilocystidia. Using the key of the Chinese species (Zeng et al. 2017), *Pulveroboletusafricanus* approaches closest to *P.brunneoscabrosus* Har. Takah. The latter has a viscid veil, reddish-brown scales and white to yellowish-white basal mycelium.

### Distribution and ecology

Both new species are endemic to tropical Africa. *Pulveroboletusafricanus* was found in Benin, Burundi, Guinea, DR Congo, Malawi, Mozambique, Togo, Zimbabwe and possibly also Zambia. It prefers regions with a pronounced wet/dry season alternance and occurs in a wide variety of woodlands, savannah woodlands and gallery forests across tropical Africa. It seems absent in the dense rainforests (Congolian region). The species is terricolous and most probably ectomycorrhizal (EcM), occurring in EcM dominated forests up to 1500 m elevation. It is difficult to ascertain if the species associates with Caesalpiniaceae (*Berlinia*, *Brachystegia*, *Isoberlinia*, *Julbernardia*) and/or with *Uapaca* (Phyllantaceae). Only *Uapaca* is well represented throughout its distribution range. In Eastern Africa (Zambezian region), it is also found under *Brachystegia* spp. and *Julbernardia* spp.; in West Africa (Soudano-Guinean region) under *Berliniagrandiflora* and *Isoberlinia* spp., Thoen and Ducousso (1989) mention it under *Uapacachevalieri* Beille. The species may also occur in Zambia (ut Pulveroboletusaff.ravenelii, fig. 1H in Watling and Turnbull 1992).

*Pulveroboletussokponianus* has so far only been found in a variety of savannah woodlands and gallery forests in the Soudano-Guinean transition zones of Benin and Togo, probably also in Ivory Coast (see fig. 3a in Léabo et al. 2017). The species is terricolous, most probably ectomycorrhizal (EcM) and most often found under *Isoberliniadoka* (Caesalpiniaceae). Since habitat destruction and felling of host trees is common practice in Benin, Yorou and De Kesel (2011) placed this species (mentioned as *P.ravenelii*) under the IUCN threat category ‘vulnerable’.

## Supplementary Material

XML Treatment for Pulveroboletus africanus

XML Treatment for Pulveroboletus sokponianus
